# Discriminant Value of Rome III Questionnaire in Dyspeptic Patients

**DOI:** 10.4103/1319-3767.77244

**Published:** 2011

**Authors:** Shahab Abid, Shaheryar Siddiqui, Wasim Jafri

**Affiliations:** 1 Department of Medicine, Aga Khan University, Karachi, Pakistan; 2 Department of Medicine, Karolinska Institute, Stockholm, Sweden

**Keywords:** Functional dyspepsia, Rome III criteria, epigastric pain syndrome, postprandial distress syndrome

## Abstract

**Background/Aim:**

Rome III criteria has modified the description of functional dyspepsia (FD) and divided this into subgroups. However, the discriminative value of Rome III questionnaire-based diagnosis of FD is yet to be determined. Objectives: To evaluate the Rome III questionnaire for the diagnosis of FD and whether it can discriminate between postprandial distress syndrome (PDS) and epigastric pain syndrome (EPS) in patients with dyspeptic symptoms.

**Patients and Methods:**

Consecutive patients, who were not on proton pump inhibitors (PPI), were asked to participate. Patients who have previously established acid peptic disease or predominantly reflux symptoms or having alarm symptoms such as weight loss and hematemesis were excluded. Rome III questionnaire for FD was used to identify the patients as having FD and divide into its subgroups; PDS or EPS. Gastro-duodenal biopsies, liver function tests and ultrasound were done to establish the diagnosis of FD.

**Results:**

Out of 272 patients with upper gastrointestinal (GI) symptoms without alarm features, who were enrolled in the study, a total of 191 (70%) fulfilled the criteria of FD based upon Rome III questionnaire. EPS subgroup was found in 109 (57%), PDS in 17 (9%) patients, overlap between EPS and PDS was present in 56 (29%) patients. Nine (5%) patients remained indeterminate. Diagnosis of FD was established in 136/191 (71%) patients only. Gastritis was present in 116 patients (85%), Duodenitis in 44 (32%) and *Helicobacter pylori* infection in 70 (51%) patients. Among 55 patients (29%) who had organic diseases, EPS was seen in 35 (64%), PDS in 5 (9%) and overlap in 15 (27%) patients. Underlying organic causes were gastric or duodenal ulcers in 14 patients, Barrett esophagus in five, chronic liver disease in seven, gall stones in five, Giardiasis and celiac disease in three each. Gastric carcinoma, Crohns disease and gastric polyps were seen in one patient each.

**Conclusion:**

This study indicates that 30% of patients who fulfilled the Rome III criteria for FD actually had organic disease. Almost one-third of patients with functioanl dyspepsia did not qualify for one of the two subgroups of FD of Rome III. There is also a need to further define the Rome III-based subgroups of FD for research purpose.

Dyspepsia is a condition of great clinical significance as a large number of population all over the world, present with dyspeptic symptoms, visit gastroenterology clinic. The prevalence of upper gastrointestinal (GI) symptoms is up to 40% in patients from the Western countries who visit GI clinics. Majority of them yield no substantial findings during endoscopy which could possibly explain their symptoms and therefore diagnosed as having functional dyspepsia (FD).[1,2] Patients with dyspepsia typically present with varied and vague symptoms, and find it difficult to discern between the subjective feeling of pain and discomfort.[3] Over the years Rome criteria have been developed to classify dyspepsia symptoms in certain group for better understanding, elaborative and much clearer picture.

Rome I committee described FD as chronic or recurrent pain/discomfort occurring in the upper abdominal region and divided it into three subgroups (i) ulcer like FD (ii) dysmotility like FD and (iii) unspecified FD.[4] The Rome II modified Rome I by sorting subtypes on the basis of predominant symptoms present in each of the subtype.[5]

The Rome III committee has subclassified FD into two main categories namely epigastric pain syndrome (EPS) and postprandial distress syndrome (PDS). Under this criterion the GI symptoms are considered to be originating from the gastro-duodenal region, the pain is centered in the epigastrium, sharp or burning in quality, the pain is intermittent, not generalized or localized to other abdominal or chest regions and not relieved by passage of stool or flatus. The term FD also encompasses early satiety and bothersome postprandial fullness which is disproportional to the size of the meal occurring several times per week.[6]

To classify the patient as having either EPS or PDS, any organic cause has to be excluded which could likely explain the symptoms; therefore, upper GI endoscopies, ultrasound and blood tests are done as this is a diagnosis of exclusion. The symptoms should be present for the preceding 3 months while the onset should be 6 months before the diagnosis.

The aim of this study was to evaluate the Rome III questionnaire for the diagnosis of FD and its usefulness to discriminate between PDS and EPS in patients with dyspeptic symptoms.

## PATIENTS AND METHODS

### Setting

Consecutive new patients who presented in the endoscopy suite of the Aga Khan University Hospital with dyspeptic symptoms were recruited; the patients were referred from the consultant clinic for upper GI endoscopy where history taking and clinical examinations were done. In addition, the patients also had complete blood count, urea, creatinine, electrolytes and liver function tests and ultrasound of upper abdomen. Patients who had established acid peptic disease by previous endoscopic examination or those with predominant heartburn, regurgitation symptoms or a weight loss of more than 5 kg in the last 6 months were not considered for possible inclusion in the study.

### Inclusion/exclusion criteria

Inclusion criteria for the study was the presence of dyspeptic symptoms for at least 3 months with the onset being at least 6 months before clinical visit, and absence of exclusion criteria included the presence of symptoms for a time period shorter then otherwise specified by the Rome III criteria, history of abdominal surgeries or finding of any organic cause during endoscopies such as gastric atrophy, erosive esophagitis, peptic ulcers and cancers. Patients using NSAIDS and long-term proton pump inhibitor (PPIs) therapy were also excluded. Patients with organic, systemic or metabolic diseases including diabetes which would otherwise explain these symptoms were also excluded. Multiple biopsies were taken during endoscopies for histological review and for absence/presence of *Helicobacter pylori*.

### Rome III FD questionnaire

Each patient was subjected to the Rome III questionnaire and the details noted by the doctor who marked the questionnaire. He was also supposed to explain the question and queries regarding the meaning of medical terms and elaboration of symptom when asked by the patients. The physician who filled the forms was blinded for the results of endoscopy and other tests. The main reason for this indirect approach was the lack of English language proficiency in the population. Interviews were done before the endoscopic examination by an experienced research officer who is well-versed with the dyspepsia questionnaire.

Endoscopies were performed by two experienced endoscopist (W.J. and S.A.) who had the experience of doing this procedure for more than 20 years. All patients underwent endoscopy and duodenal and gastric biopsies were taken in all patients. In suspected cases esophageal biopsies were also taken for excluding Barrett’s esophagus.

### Statistical analysis

All analyses were carried out by using the Statistical Package for Social Sciences (release 13, standard version, SPSS; Chicago, IL, 2004).

### Ethical approval

Informed consent was taken from each patient. The research protocol was duly approved by the ethics committee of the University Hospital.

## RESULTS

During the study period, 367 patients underwent endoscopic examination for upper GI symptoms of which 95 were excluded on the basis of exclusion criteria. A total of 272 consecutive patients, 164 (60%) males and 108 (40%) females with a mean age of 40± 14.5, were subjected to FD questionnaire.

### Diagnostic yield of Rome III criteria

Out of 272 patients, 191 (70%) fulfilled the Rome III criteria of FD before confirmation by endoscopy and laboratory investigation. PDS variant was found in 17 (9%) patients which was the least dominant of the subtypes of FD. EPS was found in 109 (57%) patients and overlap between EPS and PDS was present in 56 (29%) patients. This overlap occurred because patients fulfilled the symptoms of both these subtypes, and neither one of them was more dominant. Nine (5%) patients did not fit the Rome III criteria of either category.

**Figure 1:**
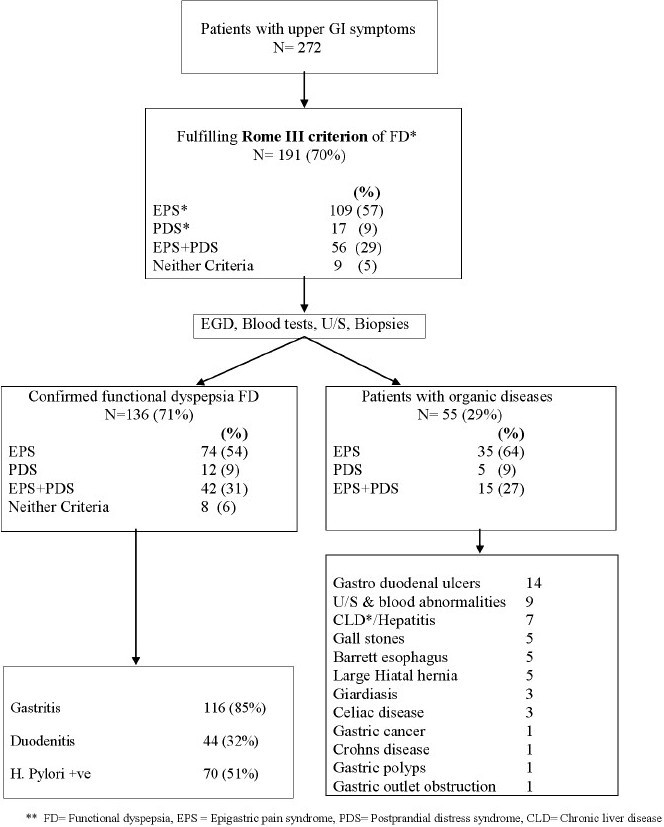
Flow chart showing the distribution of uninvestigated dyspepsia after application of Rome III questionnaire

Diagnosis of FD was established in 136 (71%) patients after excluding all organic, metabolic and systemic diseases. Organic diseases were mainly found in the EPS subgroup with 35 (64%) patients who fulfill the Rome III questionnaire criteria for FD before the investigations to rule out organic diseases, followed by the overlap group 15 (27%) patients and PDS was present in 5 (9%) patients [Figure 1].

### Prevalence of *H. pylori* in FD patients

*H. pylori* infection was found in 70 (51%) patients diagnosed with FD on gastric biopsies. Among subgroups of FD, *H. pylori* was seen in 34 (48.5%) patients of EPS, 5 (7%) patients of PDS group, and 29 (41%) patients of the overlap group and in two patients who did not fall into either category.

### Endoscopic findings

Endoscopy was normal in 136 patients, as they had no macroscopic abnormality on endoscopy which might explain their symptoms. Histopathological findings showed gastritis in 116 (85%) patients and non-specific duodenitis in 44(32%).

### Underlying organic causes among patients who fulfill Rome III questionnaire criteria for FD

Among patients who fulfilled the Rome III criteria for FD before subjecting to investigation, 55 (29%) patients had organic diseases. Among these, gastric or duodenal ulcers were seen in 14 (25.5%), chronic liver disease or hepatitis in seven (12.8%), gall stones and Barrett’s esophagus in five (9%) patients each, large hiatal hernia in another five (9%), Giardiasis and celiac disease was found in three (5.5%) patients each. Similarly gastric cancer, Crohn’s disease, gastric outlet obstruction and gastric polyps were the underlying diseases presented with dyspeptic symptoms in one patient each [Figure 1].

## DISCUSSION

FD is a heterogeneous disorder with diverse symptoms. Due to this reason the Rome committee has recommended using the term “dyspepsia symptom complex” rather than “FD” for research.[7] Rome III has tried to group several dyspeptic and dysmotility symptoms into two major groups i.e., EPS and PDS. The time interval was also made less limiting for symptoms.[6] The purpose of this subgroup formation was to categorize the patient symptoms complex more precisely and to facilitate the uniformity in defining the patients of FD for research.

Rome III classification is beneficial for researchers as it allows them to easily include and exclude patients using this criterion. However, not every patient fits perfectly into each category and therefore, results may not accurately correspond to the patient pool presenting in the clinics.[8] The present study is the direct evaluation of discriminative value of Rome III questionnaire for FD in clinical setting. Our data suggests that Rome III questionnaire for FD was able to correctly place patients with dyspeptic symptoms into the FD category two out of three times in a clinical setting when this criteria was applied to patients with uninvestigated dyspepsia. In remaining one-third patients, though the Rome III questionnaire fulfilled the criteria of FD, these patients had an organic cause for their dyspeptic symptoms. Moreover, a large number of patients cannot be categorized into either EPS or PDS subgroup and were left behind uncategorized. Documented literature has also provided evidence of significant overlap between presenting symptoms of FD. In fact a study has shown that a considerable portion of patients are not classifiable at all.[8]

In a previously published study it was demonstrated that patients fulfilling criteria for both subgroups of FD had symptoms that were psychopathologically more severe than those of patients without overlapping.[9] Ideally, detection of a specific disease marker may help clinicians make an accurate diagnosis of conditions like EPS and PDS. This would help us recognize them as separate disease entities which, currently, can only be diagnosed after ruling out everything else.[4]

Although the new criterion does allow us to categorize patients with a greater ease yet its implication in the management of patients are not very clear. The present study did not evaluate the clinical usefulness of Rome III criteria in the management of FD. However, previous studies have shown that EPS may be alleviated by using PPIs,[10,11] similarly symptoms of early satiety related to PDS improve using prokinetic drugs.[12,13] However, there is no study in the literature that have tested the effect of PPI or prokinetics in Rome III-based subgroups of patients with FD. There is need to explore the effects of specific treatment on FD subgroups on the basis of Rome III criteria. So far in research settings treatment of a syndrome based solely on symptoms has not yet provided satisfactory results.[12–14]

As FD remains a diagnosis of exclusion, there is more that needs to be done to precisely diagnose it on the basis of a defined criterion before using it for the evaluation of specific treatment outcomes and also in research. Although we had excluded patients with typical heartburn and regurgitation, still one would expect some patients to have esophagitis in the present series; however, it was surprising to note that none of the patients had erosive esophagitis on endoscopic examination. However, large hiatus hernia and Barrett’s esophagus were found in five patients each. A biopsy from distal esophagus could have further elaborated the frequency of GERD in this series of patients. Moreover, the proportion of PDS was somewhat lower than the published series of patients with FD. This could be related to the fact that it is a tertiary care setting study where the patients who have more symptoms are likely to come more than the patients with PDS. This study is conducted in a high prevalent area of *H. pylori* infection and chances for EPS are likely to be more. Therefore, it should be noted that the present study is a hospital-based study and the generalizability of this study is compromised.

In conclusion this study indicates that large number of patients did not qualify in distinct Rome III-based subgroups of FD resulting in overlap of symptoms. The Rome III criterion has neither defined any category for overlap of symptoms of EPS and PDS, nor the group of patients who do not fit in to either of the subtypes, and are left without a subgroup categorization. However, this is a hospital-based study and may not reflect the true picture of patients with FD in the primary care setting to whom the patients with FD belong. We suggest that there is a need to redefine Rome III subgroups for FD. The addition of a third mixed symptom group in the Rome III classification of FD and a possible fourth group of FD patients with indeterminate symptoms, should be considered. This will probably be more useful for assessment of outcome following interventional or exploratory research in FD patients.
